# A lactylation modification-related prediction model for the diagnosis of ulcerative colitis based on machine learning

**DOI:** 10.3389/fimmu.2026.1717232

**Published:** 2026-03-11

**Authors:** Jian Liu, Xiaoyun Kang, Yanxiang Zhou, Jiao Li

**Affiliations:** 1 Department of Gastroenterology, Renmin Hospital of Wuhan University, Wuhan, Hubei, China; 2 Department of Surgical Oncology, First Affiliated Hospital of Xi’an JiaoTong University, Xi’an, China; 3 Department of Ultrasound Medicine, Renmin Hospital of Wuhan University, Wuhan, Hubei, China

**Keywords:** lactylation, machine learning, predictive model, random forest, ulcerative colitis

## Abstract

**Background:**

Lactylation modification serves as a critical link between metabolic reprogramming and epigenetic regulation, playing a significant role in the progression of both malignant tumors and inflammatory diseases. Nevertheless, its specific function in the pathogenesis of ulcerative colitis (UC) remains poorly understood.

**Methods:**

The hub genes associated with lactylation in UC were identified and validated by mining three UC-related datasets (GSE206285, GSE75214, and GSE87466) from the GEO database, and we created a lactylation-related prediction model for the diagnosis of UC. The lactylation levels of different immune cells were also investigated via single-cell (sc) RNA-sequencing data. Finally, the core genes of lactylation were validated *in vitro*.

**Results:**

Four lactylation-related core genes (HIF1A, SLC25A12, SLC16A3, and PFKFB2) that are closely correlated with UC were identified by three machine learning methods, and the lactylation-related prediction model based on the four genes exhibited outstanding diagnostic performance for UC (AUC:0.976, 95% CI: 0.941–1.00). scRNA-sequencing analysis revealed that HSC, NK, and macrophage cells exhibited higher lactylation-related scores in UC compared to other immune cells. After Nala intervention, the expressions of the four core genes were significantly increased, while the expressions of the four genes were significantly decreased after treatment with 2-DG.

**Conclusion:**

By applying machine learning methods to analyze sequencing data, we identified core lactylation-related genes in UC and developed a diagnostic model with high predictive performance. Furthermore, based on scRNA-seq data, we investigated lactylation modifications across seven types of immune cells in UC patients, providing valuable insights into the interplay between lactylation and immune cells in UC.

## 1 Introduction

Ulcerative colitis (UC) is a relapsing inflammatory bowel disease (IBD) that occurs mainly in the colon (1). UC sufferers around the world were estimated to be at 5 million cases (2), while the global prevalence is still increasing, causing heavy health and economic burdens. The common agents for the treatment of UC include 5-aminosalicylic acid (5-ASA), corticosteroids, biologics, and selection of drugs, which mainly depends on the severity of gut inflammation (3). Fecal microbiota transplantation is an emerging treatment technology, which is considered experimental. Except for progression, UC can also give rise to some severe complications, such as bleeding, colon perforation, or even canceration. Surgical resection may be needed in the presence of severe complications of UC (4). The detailed pathogenesis of UC is currently unknown, which involves a complex interplay among genetic predisposition, environmental factors, and immune disorder (5). Therefore, an in-depth investigation into the pathogenesis of UC might help us identify the novel therapeutic targets for treating it.

Lactate, an end product of glycolysis, has traditionally been viewed as a metabolic waste (6). Recently, intracellular lactate drives lysine lactylation on histones, which is a new type of post-translational modification. Lactylation could link gene transcription regulation to epigenetic mechanisms and usually occurs in metabolically active cells (7). Mounting evidence suggest that lactylation modification plays a key role in the progression of tumors and inflammatory diseases. Liu et al. (8)reported that activation of the GCGR/GLP1R pathway could reduce intestinal fibrosis in Crohn’s disease (CD) via the regulation of histone H3 lactylation modification. However, few studies reported the role of lactylation modification in the progression of UC.

Lactylation modification has already been established as a hallmark for diagnosing some diseases. Sun et al. indicated that lactylation modification became a potential indicator for diagnosis of sepsis and guiding the treatment of sepsis (9). Zhang et al. accurately predicted the prognosis of individuals with colorectal cancer based on a 23-gene lactylation-related gene risk model (10). Wu et al. created the six lactylation-related genes model to differentiate CD tissues from normal tissues, suggesting that the lactylation-related gene model could serve as the diagnostic tool for CD (11). Currently, no researchers have reported a diagnostic model for UC based on lactylation-related genes, and the selection of lactylation-related genes in UC might help in the early diagnosis of UC and in the design of novel therapeutic agents.

Given that abnormal lactylation modification has been identified in the progression of UC (12), there is an urgent need to precisely diagnose UC based on the lactylation-related gene risk model. Therefore, the present study applied integrated bioinformatic analysis based on multiple public sequencing data to determine the hub genes for lactylation modification in UC. Then, we used three machine learning methods to create the lactylation-related gene risk model. This study provided a novel approach to identify lactylation-related biomarkers for the diagnosis of UC and also new molecular targets for treating UC.

## 2 Methods

### 2.1 Dataset sources and data processing

Three UC-related gene expression datasets (GSE206285, GSE75214, and GSE87466) from the GEO database were applied in the subsequent analyses (550 UC patients and 18 healthy individuals from GSE206285, 74 UC patients and 11 healthy individuals from GSE75214, and 87 UC patients and 21 healthy individuals from GSE87466). The expressions of significant genes between UC individuals and healthy controls were compared using the “ggplots2”, “limma”, and “heatmap” packages.

### 2.2 Functional enrichment analysis

Functional enrichment was performed using the Metascape database, which provides a detailed resource for annotating gene lists. Functional enrichment analysis was conducted to evaluate the biological processes of the hub genes, including GO and KEGG analysis.

### 2.3 Analysis of single-cell RNA sequencing data

We obtained a UC-related scRNA-seq data from the GEO database (GSE162335) containing 18 UC individuals. We eliminated batch effects among UC samples using “harmony” package and then normalized the scaled data using the ScaleData function. Uniform manifold approximation and projection (UMAP) was used for data mining. Principal component analysis (PCA) was also used to reduce the dimensions on the scaled data. We employed the FindAllMarkers function to identify differential genes in each cluster, and cluster annotation was performed based on marker genes. LRG scores were calculated using the AUCell package, which was reported in a previous study (10).

### 2.4 Machine learning algorithms

Three machine learning methods were used in the present study to identify core genes of lactylation. LASSO regression, support vector machine (SVM), and random forest algorithms were reported in our recent research (13). LASSO regression was executed using the “glmNETs” R package. Random forest classifier was implemented using the “randomForest”package, and SVM was implemented with”kernlab” R packages.

### 2.5 Blood collection

Blood samples were collected from three UC individuals and three healthy individuals. After centrifuge, the plasma was frozen at -80 °C in a refrigerator. The six individuals all gave their informed consent of this medical research, and this research was successfully passed by the Medical Ethics Committee of Renmin Hospital of Wuhan University [No: 2022K-K265 (Y01)]. All of the individuals gave their informed consent. This research was performed in line with the Declaration of Helsinki.

### 2.6 Cell culture and intervention

NCM460 (the human intestinal epithelial cell line) and RAW264.7 (a mouse-derived macrophage cell line) were bought from the Chinese Academy of Sciences (Shanghai, China). The two cell lines were authenticated for the following experiments and have not been previously reported as misidentified, and they are free of mycoplasma contamination. Cell lines were cultured in RPMI-1640 culture medium mixed with 10% fetal bovine serum. The cell lines were cultured in a humidified atmosphere filled with 5% CO_2_. The cellular experiments were divided into four groups: normal control, LPS, LPS + Nala, and LPS + 2-DG. The cells were treated with 1 µg/mL LPS, 10 mM Nala (yuanye, Shanghai, No. R32899), and 10 mM 2-DG (Aladdin, CAS: No.D109194), respectively.

### 2.7 Real-time quantitative PCR

Total cellular RNA was extracted using the TRIzol method. After quantitation and purification, the total RNA was reverse-transcribed, and qPCR was performed using SYBR Green qPCR Kits (ELK Biotechnology,EQ001). The expression levels of the target genes (HIF1A, SLC25A12, SLC16A3, and PFKFB2) were normalized with GAPDH, and the detailed primer sequences are shown in **Supplementary Table 1**.

### 2.8 Western blot

The total proteins were abstracted from the two cell lines, and the protein concentration was quantified with BCA protein assay kit. The cell lysates were first boiled for 10 min, separated by using SDS-PAGE, and then transferred to polyvinylidene fluoride (PVDF) membranes. The PDVF membranes were blocked with 5% milk for 1 h and then incubated with primary antibodies (HIF1α: 1:1,000, affinity, no. AF1009; SLC25A12: 1:1,000, AB clonal, no. A21129; SLC16A3: 1:1,000, Proteintech, no. 22787-1-AP; PFKFB2:1:1000, Proteintech, no. 30425-1-AP). After being washed with PBS, the membranes were incubated with appropriate secondary antibodies. Finally, the bands were exposed to film, and β-actin was utilized as a reference protein.

### 2.9 Statistical analysis

Statistical analysis was carried out using SPSS (v20.0) and R software (v4.1.2). Receiver operating characteristic (ROC) curve was used to determine the diagnostic value of the lactylation-related prediction model. Decision curve analysis (DCA) plot was drawn to assess the clinical utility of the lactylation-related prediction model. Student’s *t*-test was employed to compare the differences between two groups for continuous data. *P*-value less than 0.05 was defined as statistically significant.

## 3 Results

### 3.1 Identification of hub genes associated with UC

**Figure 1:**
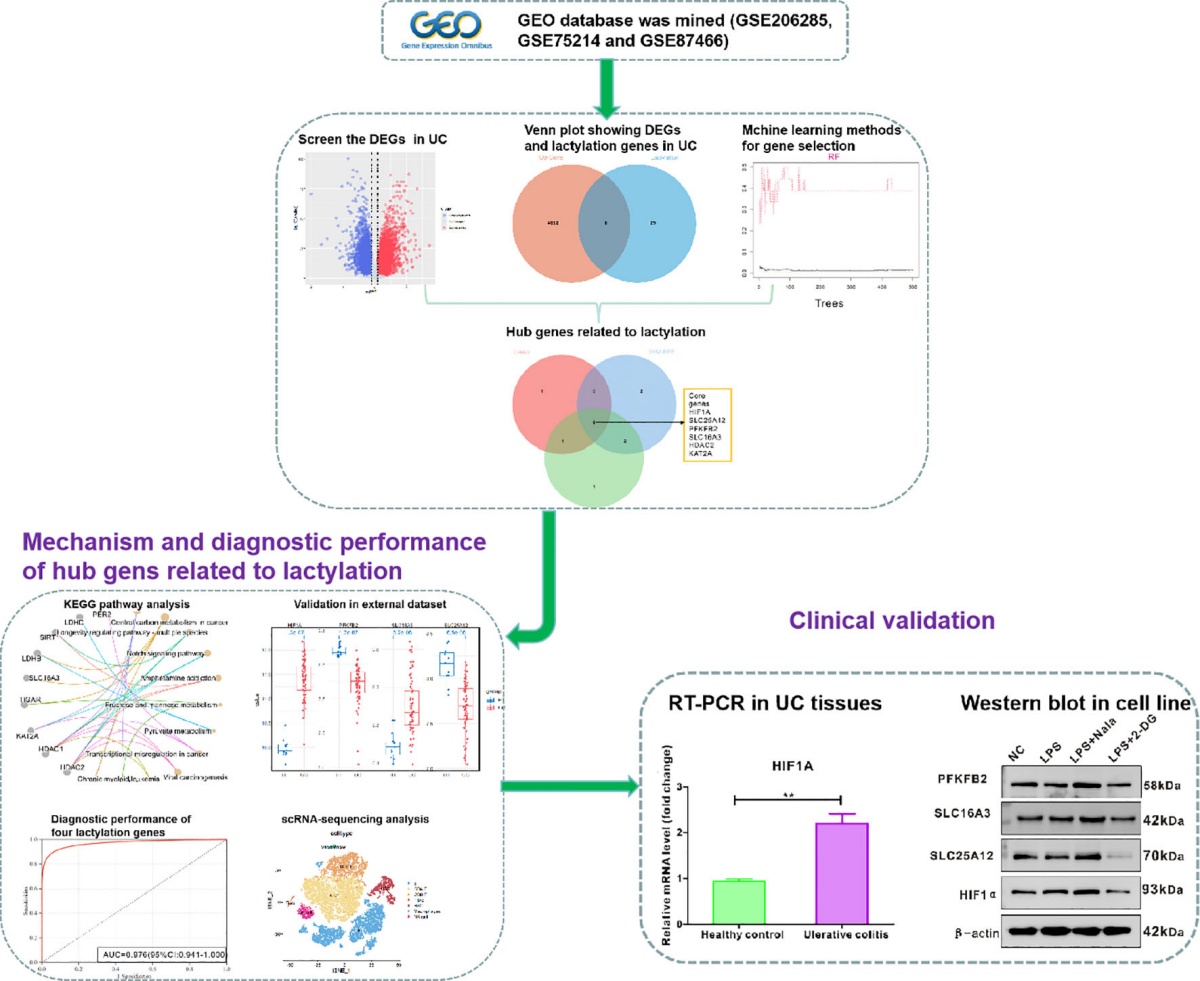
Systematic flowchart of this study.

**Figure 2:**
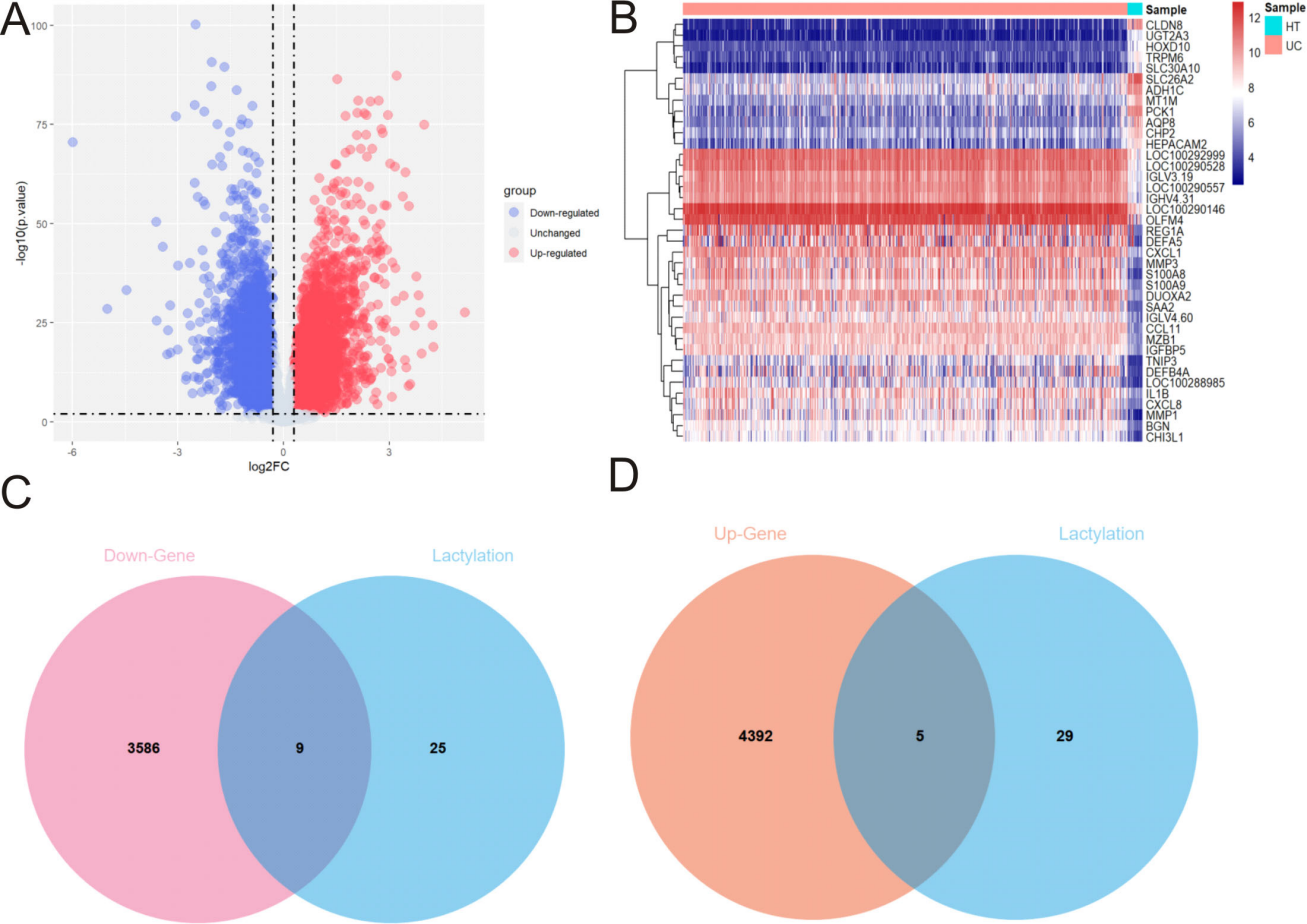
Selection process of the hub genes of lactylation in UC. **(A)** Volcano plot showing the downregulated and upregulated genes based on GSE206285. **(B)** Heat map displaying the significant genes between UC and control tissues. Venn diagrams show the downregulated genes **(C)** and upregulated genes with lactylation in UC **(D)**. UC, ulcerative colitis.

The systemic flowchart of this analysis is shown in **Figure 1**. GSE206285 was selected as the training set, which included 550 cases of UC individuals and 18 cases of healthy controls. The volcano map shows the downregulated genes (*N* = 3,595) and upregulated genes (*N* = 4,397) between the UC tissues and normal tissues (**Figure 2A**). The heat map displays the expression profiles of significant genes in UC tissues and normal tissues (**Figure 2B**). A total of 34 lactylation-related genes from gene ontology reported in a recent bioinformatic research (13) were used to intersect with differential genes. The Venn plots revealed that there are nine lactylation-related genes with downregulation (**Figure 2C**) and five lactylation-related genes with upregulation (**Figure 2D**). In brief, we identified 14 key genes associated with lactylation in UC.

### 3.2 KEGG enrichment analysis

The above-mentioned significant genes in UC were subjected to functional analysis. GO analysis revealed that these genes were mostly involved in lactate metabolic process, glucose metabolic process, histone deacetylation, histone deacetylase complex, and histone deacetylase activity (**Supplementary Figures 1A–C**). Further KEGG analysis revealed that these genes were enriched in longevity-regulating pathway, notch signaling pathway, pyruvate metabolism, and thyroid hormone signaling in UC. The correlations between lactylation-related genes and KEGG pathways are shown in **Supplementary Figure 1D**.

### 3.3 Selection of lactylation-related genes in UC via machine learning

**Figure 3:**
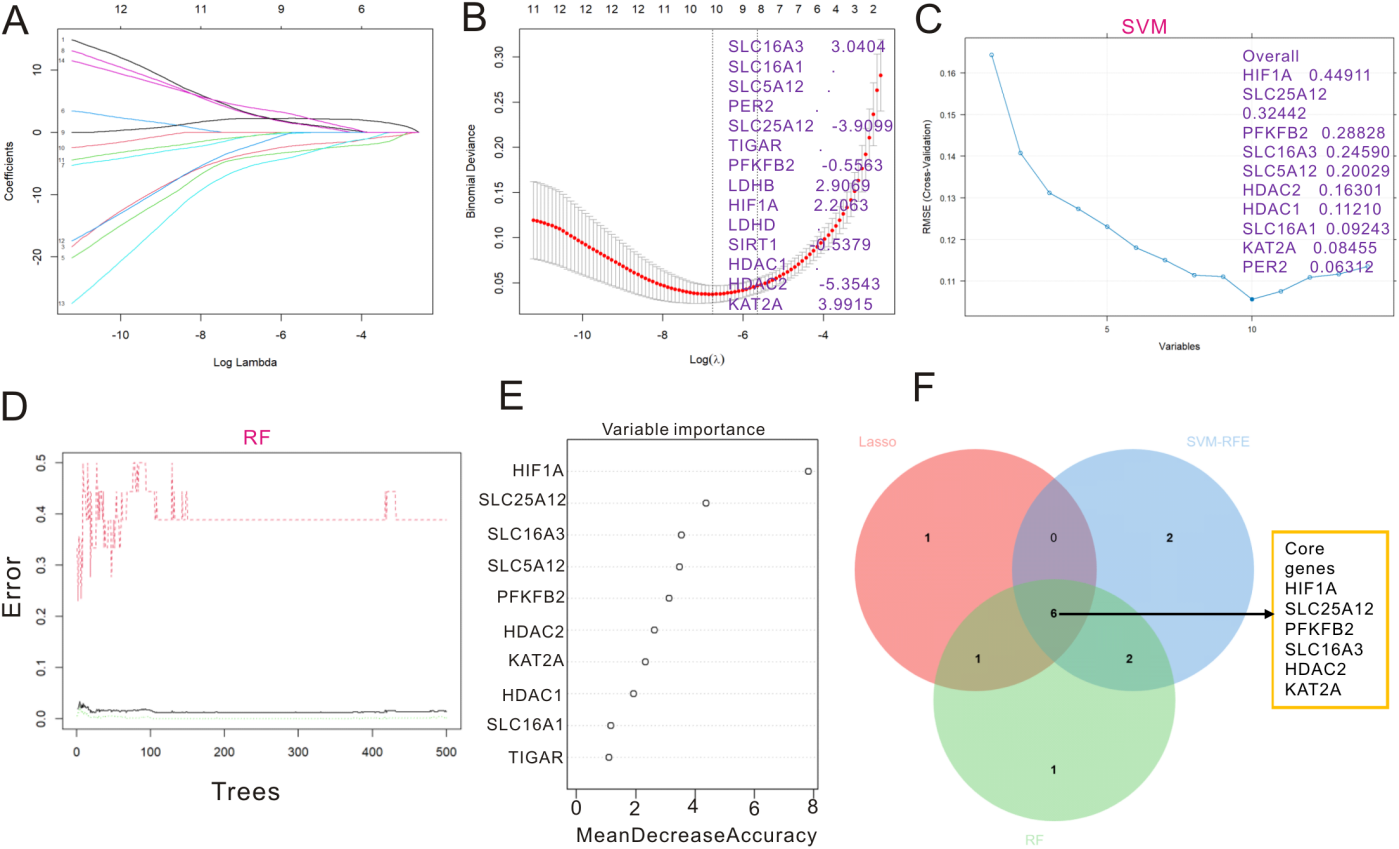
Core genes with lactylation were identified by three machine learning methods. **(A)** Distribution of hub LRGs in 10-fold cross-validation of LASSO. **(B)** LASSO regression coefficient distribution for LRGs. **(C)** A total of 10 significant genes with lactylation were identified by SVM. **(D)** A total of 10 key genes with lactylation were identified by RF. **(E)** Importance of key genes with lactylation ranked by RF. **(F)** Venn plot displaying the overlap genes of lactylation selected by three machine learning methods. LRGs, lactylation-related genes; SVM, support vector machine; RF, random forest.

In order to screen key genes of lactylation, three machine learning algorithms were applied in the selection process. LASSO regression identified eight key genes of lactylation (**Figures 3A, B**), and both SVM-RFE (**Figure 3C**) and random forest (**Figure 3D**) algorithms identified nine key genes of lactylation in UC. The rank of each gene selected by RF is listed in **Figure 3E**. The Venn plot was drawn to show the overlapping genes from three machine learning methods, and six genes were selected as the key genes of lactylation, including HIF1A, SLC25A12, SLC16A3, PFKFB2, HDAC2, and KAT2A (**Figure 3F**).

### 3.4 Validation of the hub genes with other datasets

**Figure 4:**
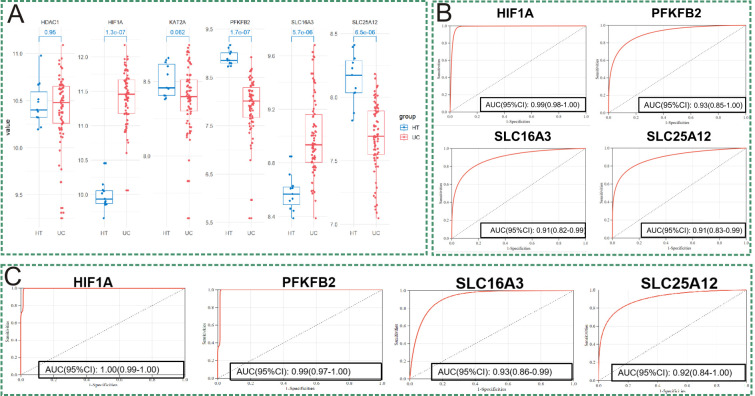
Validation of the key genes with lactylation in GSE75214. **(A)** The expression profiles of six key genes with lactylation in UC and only four genes (HIF1A, SLC25A12, PFKFB2, and SLC16A3) were significantly different between the UC and normal tissues. **(B)** Predictive performances of the four core lactylation-related genes for the diagnosis of UC in GSE206285. **(C)** Validating the predictive performances of the four core lactylation-related genes with GSE75214.

Another UC cohort (GSE75214), which included 74 cases of UC individuals and 11 cases of healthy controls, was used to validate these key genes of lactylation. As shown in **Figure 4A**, the expression of HIF1A and SLC16A3 were upregulated in UC tissues, and the expressions of PFKFB2 and SLC25A12 were downregulated in UC tissues compared with the controlled tissues, while there was no significant differences of HDAC2 and KAT2A between UC tissues and the controlled tissues. Hence, HIF1A, SLC25A12, SLC16A3, and PFKFB2 were finally identified as the hub genes of lactylation in UC. Subsequently, the ROC curves indicated that each of the hub genes of lactylation could well differentiate UC individuals from the healthy controls in GSE206285 (**Figure 4B**). When validating the diagnostic performance of the hub genes of lactylation with GSE75214, the diagnostic accuracy was still robust (**Figure 4C**).

### 3.5 Predictive performance of the lactylation-related model

**Figure 5:**
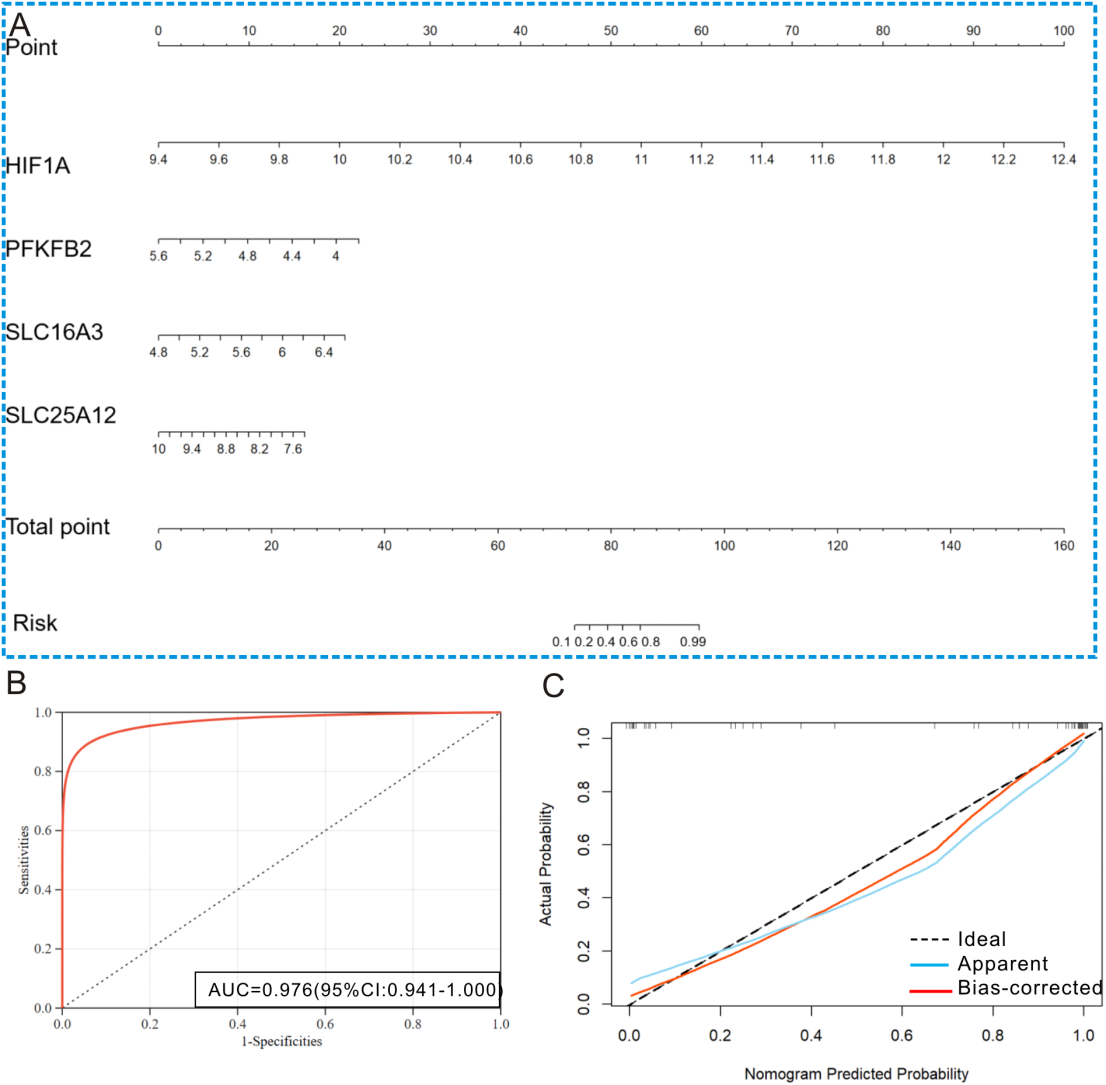
Predictive performance of the lactylation-related model to differentiate UC from control. **(A)** Diagnostic nomogram based on the four lactylation-related genes. **(B)** ROC curve showing that the lactylation-related model could well differentiate UC from control (AUC: 0.976). **(C)** DCA curve displaying that the lactylation-related model has good clinical utility.

To enhance clinical applicability, we developed a diagnostic nomogram based on four lactylation hub genes to distinguish UC patients from healthy controls. GSE87466 is a UC dataset including 87 cases of UC individuals and 21 cases of healthy controls. Based on the UC dataset, the diagnostic nomogram is displayed in **Figure 5A**. The ROC curve analysis (**Figure 5B**) demonstrated that the four lactylation-related prediction model achieved nice diagnostic accuracy, with an AUC of 0.976 (95% CI: 0.941–1.00). The DCA curve indicated that the four lactylation-related prediction models possessed an outstanding clinical applicability value (**Figure 5C**). In a word, the four-lactylation-related prediction model could be used as a diagnostic tool for UC.

### 3.6 Correlation between lactylation-related genes and immune cells via scRNA-seq analysis

**Figure 6:**
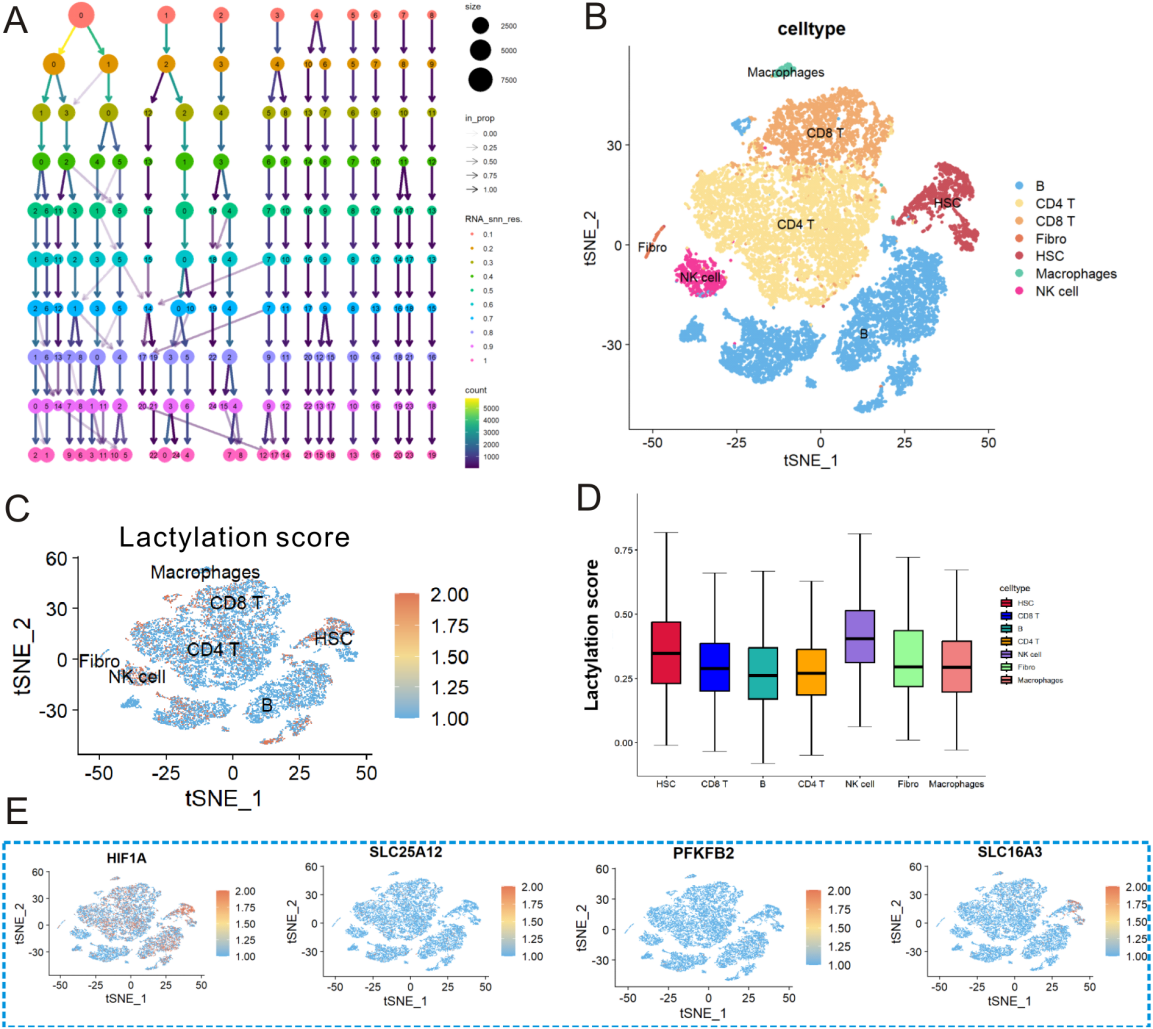
scRNA-seq analysis revealing the correlations between the four lactylation-related genes and immune cells in UC. **(A)** Cluster tree plot showing cell grouping at different resolutions. **(B)** The cells were divided into seven separate clusters. **(C, D)** Lactylation score of each immune cell in UC. **(E)** Expression profiles of the four lactylation-related genes in seven kinds of immune cells in UC.

A scRNA-seq dataset (GSE162335), including 18 cases of UC tissues, was selected for further analysis. After data cleansing, UMAP was used to reduce dimensions (**Figure 6A**) and sort cells into seven distinct clusters (**Figure 6B**). The correlations between lactylation-related genes and immune cells are displayed in **Figure 6C**. Notably, the expression of HIF1A and SLC16A3 was strongly associated with the infiltration of HSC. Then, we used lactylation-related score to assess the lactylation level of each immune cells in UC. As exhibited in **Figure 6D**, the lactylation-related score was different among the different immune cells, and NK cells, HSC, and macrophage cells exhibited a higher lactylation-related score in UC. Finally, the detailed expressions of the four key genes of lactylation in seven types of immune cells are shown in **Figure 6E**.

### 3.7 Validating the four lactylation-related genes in UC

**Figure 7:**
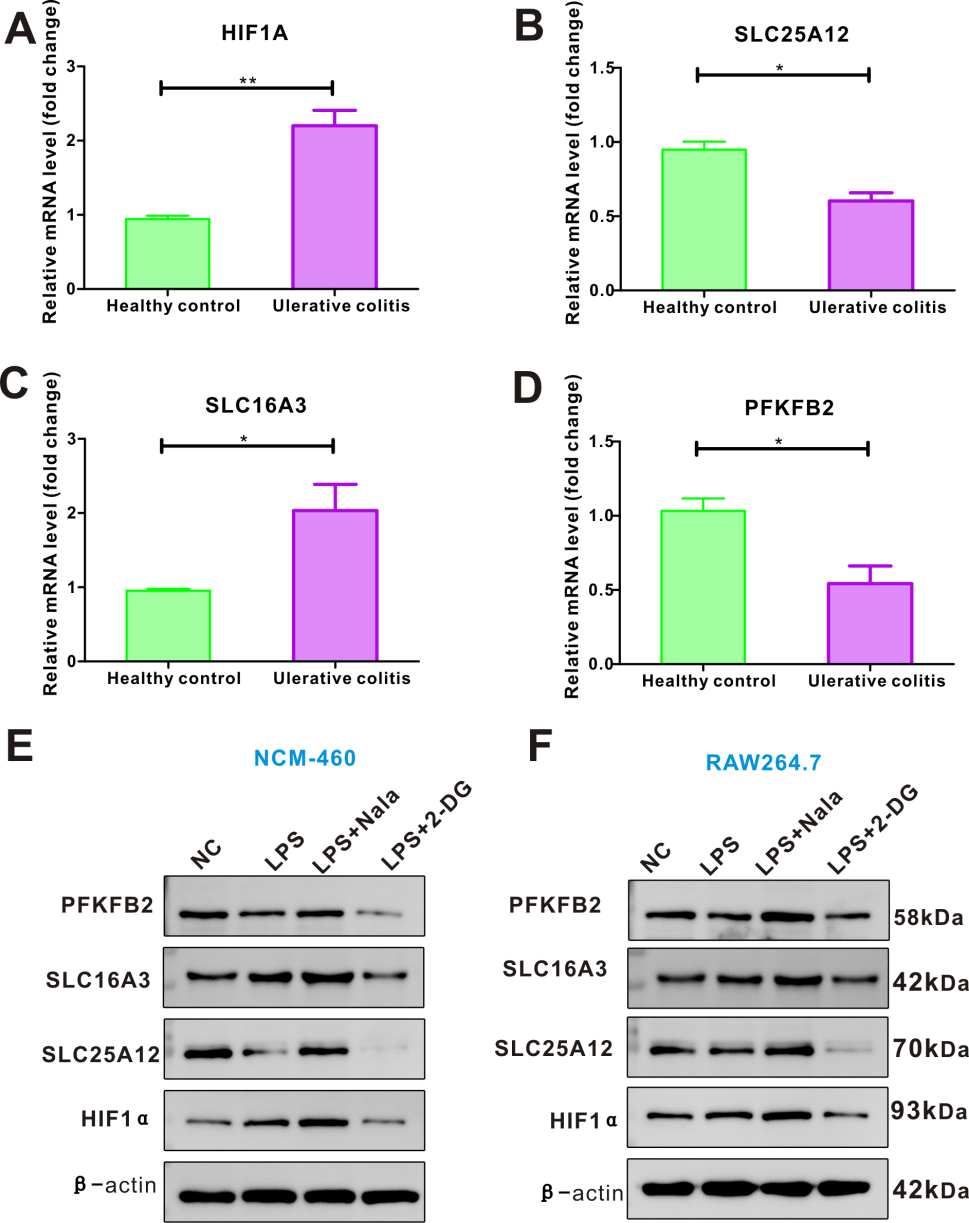
Validating the expression profiles of the four lactylation-related genes in blood samples and *in vivo*. **(A–D)** PCR analysis revealing that the expression of HIF1A and SLC16A3 mRNA were highly expressed, while the levels of SLC25A12, and PFKFB2 mRNA were downregulated in UC compared to the healthy individuals. The expressions of HIF1α, SLC25A12, SLC16A3, and PFKFB2 were significantly increased after Nala intervention, while the expressions were significantly decreased after treatment with 2-DG in NCM-460 cell line **(E)** and in RAW264.7 cell line **(F)**. *P < 0.05, **P < 0.01.

We collected blood samples from three cases of UC individuals and three healthy individuals, and RT-PCR assay measured the relative expression profiles of the four hub genes of lactylation. As shown in **Figures 7A–D**, the expression of HIF1A and SLC16A3 were highly expressed in UC compared to the healthy individuals. By contrast, the levels of SLC25A12, and PFKFB2 mRNA were downregulated in UC compared to the healthy individuals. As the four hub genes were lactylation-related genes, we determined the changes of proteins after Nala or 2-DG intervention *in vitro*. After Nala intervention, the expressions of HIF1A, SLC25A12, SLC16A3, and PFKFB2 were significantly increased. The expressions of HIF1A, SLC25A12, SLC16A3, and PFKFB2 were significantly decreased after treatment with 2-DG both in NCM-460 (**Figure 7E**) and RAW264.7 (**Figure 7F**). In conclusion, the four core genes were regulated by lactylation modification in UC.

## 4 Discussion

In this study, we analyzed multiple RNA sequencing datasets to identify hub genes closely associated with lactylation modification in UC. Using sc-RNA seq data, we further characterized the interactions between four key lactylation−related genes and immune cells. Our results reveal that lactylation levels are closely linked to abnormal immune cell activation, with lactylation predominantly observed in NK cells and macrophages. The predictive model built from these four lactylation-related genes demonstrated strong diagnostic performance, suggesting its potential utility as a diagnostic tool for UC.

UC is a chronic bowel disease featuring diffuse inflammation of the colon, and it severely lowers the patient’s quality of life. Currently, from the perspective of immunology, the gut inflammation of UC is mainly originated from the activated macrophages (14). The macrophage is a key member of the innate immune system and can secrete cytokines and kill pathogens. Due to their active metabolism, macrophages are one of the most common cell types to undergo lactylation modification (15, 16). Lactylation modification mostly occurs in tumor-associated macrophages in some cancers (17, 18), while no study has reported this similar phenomenon in UC. Our study is the first literature to report that lactylation modification is mainly occurs in macrophages based on scRNA-seq data. Moreover, we found that lactylation modification also occurs in NK cells. A recent study demonstrated that regulating lactylation modification promotes the cytotoxicity of NK cells in cancer (19), indicating that NK cell also plays a vital role in lactylation modification.

As more and more sequencing data continue to be made public, we can more easily use these data to investigate the genetic factors of disease. Machine learning is better suited to handle these massive volumes of sequencing data. With the advent of Deepseek, the application of artificial intelligence in the medical field is particularly significant (20). Li et al. used three machine learning methods (random forest, XGBoost, and Lasso regression) to develop an asparagine metabolism immunity index for prognostic evaluation (21). In our previous study, we used LASSO, random forest (RF), and support vector machine-recursive feature elimination (SVM-RFE) model to identify hub genes associated with neutrophil extracellular traps. In this study, we also employed the same three machine learning algorithms to screen the key genes associated with lactylation modification and identified HIF1A, SLC25A12, SLC16A3, PFKFB2, HDAC2, and KAT2A as the key genes in UC. After validating with other sequencing data, only HIF1A, SLC25A12, PFKFB2, and SLC16A3 still remained significant. Based on the four genes, the lactylation-related model provided nice performance for the diagnosis of UC with AUC of 0.976. Hence, machine learning algorithms identified four key genes associated with lactylation modification in UC, which might be therapeutic targets against UC.

HIF1A was identified as one of lactylation-related genes in UC in the present study. HIF1A encodes the protein of HIF-1α, which is a heterodimer composed of both α and β subunits. HIF-1A serves as a master regulator of homeostatic response to hypoxia and plays a vital role in the pathophysiology of ischemic disease and tumor angiogenesis (22). Hypoxia and genetic alterations could upregulate the expression of HIF-1A, which has been correlated with unsatisfactory prognosis in some cancer types (23). HIF-1A stimulates the over-expression of glycolytic transporters and activation of enzymes involved in glycolysis, which is the major source of lactic acid (24). Li et al. reported that HIF1A was highly expressed in experimental colitis samples compared to normal controls, and HIF1A could exacerbate intestinal inflammation in DSS-induced colitis (25). In line with their findings, the expression of HIF1A was also upregulated in UC tissues both in GSE206285 and GSE75214. Hence, we infer that HIF1A might promote the progression of UC via abnormal lactylation.

PFKFB2 is a key glucose metabolism regulator gene, which could affect cellular energy metabolism and biosynthesis by regulating key enzyme activities in glycolysis and gluconeogenesis pathways. Schilperoort et al. discovered that PFKFB2 promotes efferocytosis-induced macrophage glycolysis in a lactate-dependent manner (26). Our study found that PFKFB2 was downregulated in UC and identified PFKFB2 as a novel lactylation-related gene in UC. Both SLC25A12 and SLC16A3 belong to solute carrier family. Shen et al. demonstrated that the silence of SLC16A3 reduced the levels of extracellular lactate and alleviated hypoxia in hepatocellular carcinoma (27). A bioinformatic analysis revealed that SLC16A1 was a hub gene of lactylation in CD (15).

This research has certain limitations. The reliance on public databases related to UC without independent multicenter validation may affect the generalizability of our conclusion. Furthermore, experimental validation of lactylation was conducted only *in vitro*; future animal studies and deeper mechanistic investigations of lactylation modification related to hub genes in UC are needed. Despite these limitations, our work provides a foundational framework for future research in this area.

## 5 Conclusion

Using machine learning to analyze sequencing data, we identified key lactylation−related genes and constructed a diagnostic model. Furthermore, based on sc-RNA sequencing data, we characterized lactylation modifications across seven immune cell types in individuals with UC, offering new insights into the interplay between lactylation and immune cells in this disease. Our study provides valuable insights to identify core biomarkers and guide the future design of therapeutic targets to improve the outcomes for UC patients.

## Data Availability

The datasets presented in this study can be found in online repositories. The names of the repository/repositories and accession number(s) can be found in the article/**Supplementary Material**.
