# Supplementary material for: A lactylation modification-related prediction model for the diagnosis of ulcerative colitis based on machine learning
Source: Front Immunol. 2026 Mar 11;17:1717232. doi: 10.3389/fimmu.2026.1717232 (PMC13013276; doi:10.3389/fimmu.2026.1717232)
Supplement: Supplementary file 1 [file Table1.doc]

**Table S1**. The sense and inverse sequences of the selected primers

| Gene | Sense sequence | Inverse sequence |
| --- | --- | --- |
| HIF1A | 5'-GAAAGCGCAAGTCCTCAAAG-3' | 5'-TGGGTAGGAGATGGAGATGC-3' |
| SLC25A12 | 5'-TCTTCGGGCTCATCTTCATC-3' | 5'-GTCAGCATCCAGGTCATCCA-3' |
| SLC16A3 | 5'-CCTGCTGGTCATCATCTTCG-3' | 5'-GCCACAGCCAAAGTCAAAGA-3' |
| PFKFB2 | 5'-TCTTGCTGTGGCTCTCTGCT-3' | 5'-AACCTGCATCTCCACCTTCC-3' |
| GAPDH | 5'-GAAGGTGAAGGTCGGAGTC-3' | 5'-GAAGATGGTGATGGGATTTC-3' |


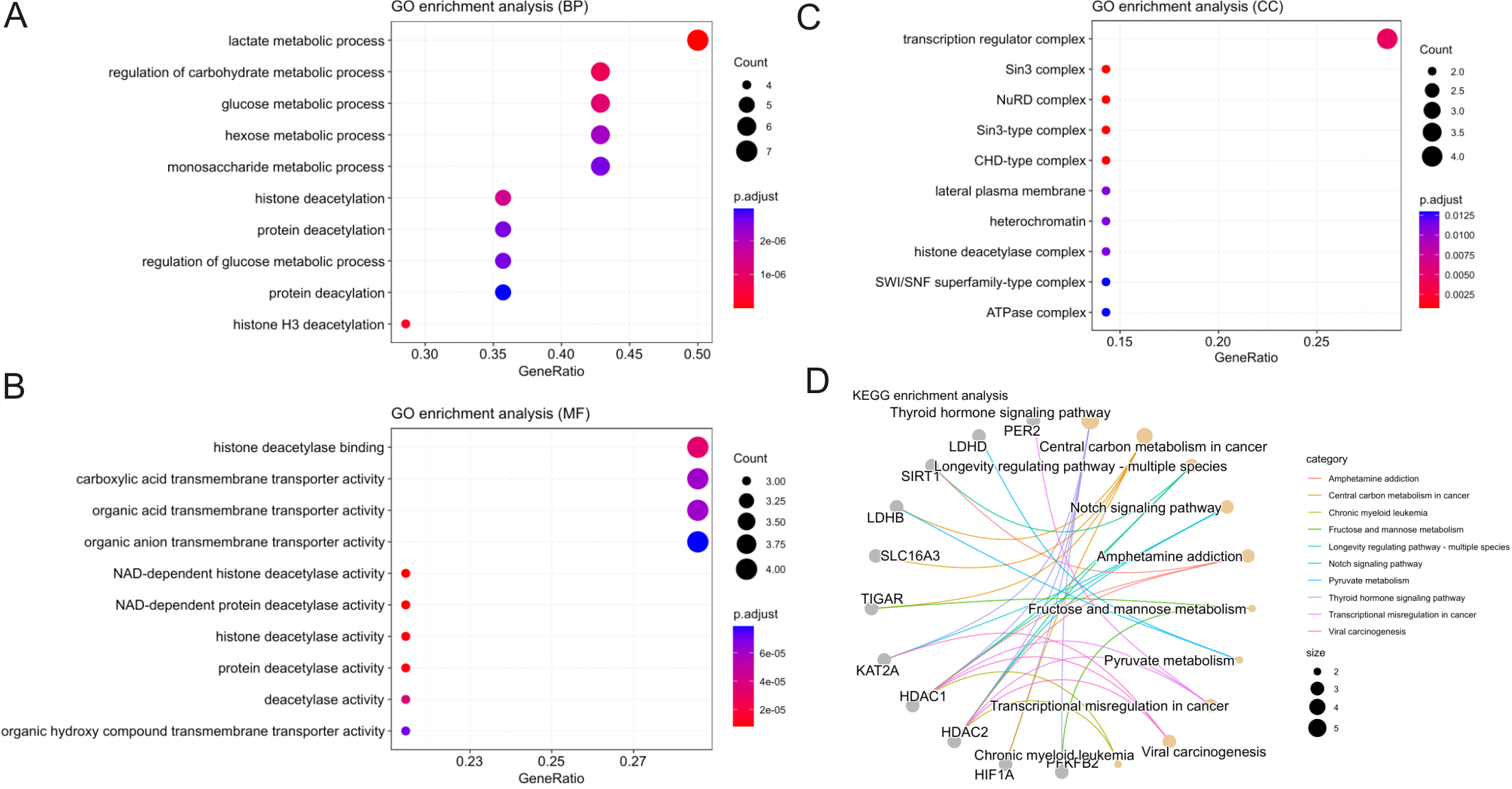


**Figure S1**. Enrichment analysis of the significant genes with lactylation in UC. **A-C.** Go analysis. **D**. Association between the lactylation-related genes with KEGG pathways.
